# Comparative, double-blind, controlled study of intra-articular hyaluronic acid (Hyalubrix^®^) injections versus local anesthetic in osteoarthritis of the hip

**DOI:** 10.1186/ar2875

**Published:** 2009-12-09

**Authors:** Alberto Migliore, Umberto Massafra, Emanuele Bizzi, Francesca Vacca, Severino Martin-Martin, Mauro Granata, Andrea Alimonti, Sandro Tormenta

**Affiliations:** 1 Operative Unit of Rheumatology, San Pietro Fatebenefratelli Hospital, via Cassia 600, 00189 Rome, Italy; 2 Department of Internal Medicine, Regina Apostolorum Hospital, via San Francesco 50, 00041 Albano Laziale, Rome, Italy; 3 Operative Unit of Rheumatology, San Filippo Neri Hospital, via Giovanni Martinotti 20, 00135 Rome, Italy; 4 Department of Radiology, San Pietro Fatebenefratelli Hospital, via Cassia 600, 00189 Rome, Italy

## Abstract

**Introduction:**

Comparison of intra-articular bacterial-derived hyaluronic acid (Hyalubrix^®^) (HA) with local analgesia (mepivacaine) for osteoarthritis (OA) of the hip.

**Methods:**

A pilot prospective, double-blind, 6-month randomized trial of 42 patients with hip OA. HA or mepivacaine was administered twice (once a month) under ultrasound guidance. Efficacy measurements included the Lequesne's algofunctional index, a visual analog scale for pain, concomitant use of analgesia, patient and physician global measurement, and safety.

**Results:**

Patients in the HA group exhibited a significantly reduced Lequesne's algofunctional index 3 and 6 months after treatment (*P *< 0.001) and significantly reduced visual analog scale pain scores 3 and 6 months after treatment (*P *< 0.05) compared with the local anesthetic group. All primary and secondary measures were significantly improved versus baseline, but other than the above were not different from each other at 3 or 6 months. Adverse effects were minimal.

**Conclusions:**

This comparative study suggests a beneficial effect and safety of intra-articular HA in the management of hip OA.

**Trial registration number:**

ISRCTN39397064.

## Introduction

Osteoarthritis (OA) is the most common joint disorder; with a calculated prevalence of 10 to 18% in the United States [1]. OA of the hip is second to the knee in frequency, with prevalence in white men of 17% and in white women of 9%, for those 60 years or older [1,2]. Within 10 years of the onset of hip OA, 30 to 50% will result in total hip replacement. As reported in the studies, 'The National Arthritis Data Workgroup reviewed data from available surveys, such as the National Health and Nutrition Examination Survey series. For overall national estimates, we used surveys based on representative samples' [1,2]. In these cited studies, clinical and radiological parameters were observed for extracting numerical data about the prevalence of OA.

Contemporary management of hip OA is directed at pain control, improvement function and improved health-related quality of life. Management of hip OA includes nonpharmacological modalities (patient education, exercise, assistive devices, and weight management) and pharmacological treatments ranging from oral to intra-articular (IA) therapy. Oral therapy consists of analgesics and nonsteroidal anti-inflammatory drugs (NSAIDs), both with attendant risks [3-6]. There is limited information on IA therapy, such as depocorticosteroids [7] and hyaluronate (hyaluronic acid (HA), hyaluronan) [8].

In addition to establishing the safety of HA treatment, many studies on OA of the knee have demonstrated good efficacy profiles for IA HA treatments [9-12]. IA HA appears to be an ideal treatment for patients with knee OA with persistent pain that does not respond to conservative treatment, or for patients that are not possible candidates for joint replacement due to clinical conditions that make surgical replacement impossible to perform. Although IA HA generally has a slower onset of action than IA steroids, the therapeutic effect of IA HA injection appears to last longer [13].

Hyalubrix^® ^is a sterile nonpyrogenic solution of HA sodium salt (15 mg/ml sodium HA) with a molecular-weight range of 1,500 to 3,200 kDa. Although biochemically processed similar to Hyalgan^®^, it is produced by bacterial fermentation. It has been used in the treatment of knee OA, and a postmarketing study on 1,523 patients supported the efficacy and safety of Hyalubrix^® ^in pain reduction and functional improvement in the knee [14].

In comparison with HA, IA local anesthetics (for example, mepivacaine) are analgesic with potential therapeutic properties such as dilution of proinflammatory cytokines in pathological synovial fluid and reduction of neuro-mediated inflammation by the inhibition of neuropeptide production. These latter actions appear to last longer than the analgesic effect [15-17].

Our previous studies [18,19], as well as other studies [20,21], suggested good efficacy profiles for IA HA treatments in hip OA as well as good safety profiles for such treatment when performed under ultrasound guidance.

The present pilot study compared the benefit, duration and adverse event profile of IA Hyalubrix^® ^versus IA mepivacaine, in the treatment of hip OA using ultrasound guidance to ensure IA injection [18].

## Materials and methods

### Study design

The study was a single-site, prospective, randomized, double-blind, controlled clinical trial of the efficacy and safety of ultrasound-guided IA HA (Hyalubrix^®^, Fidia S.p.A, Padova, Italy) versus an IA analgesic (Mepivacaine, Fidia S.p.A, Padova, Italy) in outpatients with hip OA once a month for two injections. The patients and examiner were not aware of the injected drug. A separate unblinded investigator performed the injections.

The study protocol, including informed consent documentation, insurance, and Summary of Product Characteristics, was approved by the hospital ethics committee and the study followed the guidelines of the Declaration of Helsinki.

All patients were examined at baseline, completed the Lequesne algofunctional index (grades 1 to 4), recorded hip pain on a 10 cm visual analog scale (VAS), and recorded a patient's global assessment score for hip OA. The translation of the question asked was: 'How important do you think your hip osteoarthritis is for your global quality of life?'. A physician's global score was also assessed at baseline (how important the physician thinks the hip OA is for global life quality of the patient, with a range of 0 to 10). Baseline information included NSAID consumption by the number of days in the prior month in which NSAIDs were used (range 0 to 30), plus the number of NSAIDs taken per day.

Consecutive patients with hip OA were randomly assigned to receive Hyalubrix^® ^or mepivacaine by a computer-generated block randomization schedule. The study included 42 patients, 20 women and 22 men.

Inclusion criteria included the following: age >40 years; ambulant without assistance; hip OA by American College of Rheumatology radiographic criteria; baseline VAS ≥ 4 cm; persistence of hip pain for at least 1 month before baseline; and signed informed consent.

Exclusion criteria were as follows: comorbidities (for example, rheumatoid arthritis, avascular necrosis, fibromyalgia); infection around the injection site; treatment with oral, parenteral, or IA steroids within 3 months; use of anticoagulants or history of thrombocytopenia; allergy to local anesthetics; history of adverse reaction to IA HA; pending hip replacement surgery; and use of a purported OA disease-modifying agent.

The primary endpoint of the present study was the determination of the change in the Lequesne index of the hip, comparing IA HA with IA mepivacaine at 26 weeks. Secondary endpoints were pain intensity (recorded on a 10 cm VAS), patient record of NSAID consumption, patient's global assessment, examining physician's global assessment, demographic correlations to response, and HA safety.

All adverse events were recorded with a question of severity, in relation to therapy and graded by severity. Measurements were recorded at screening, baseline, 3 months after the first injection, and 6 months after the first injection. Safety information was collected and reported on the basis sponsor's standard operating procedures, the international conference on harmonization of good clinical practice and the applicable Italian regulatory requirements.

### Treatment

IA hip injections were guided by ultrasound using an antero-sagittal approach [18,19]. The assistance of real-time ultrasound and Doppler imaging avoided vessel injection and validated penetration of the hip joint. Ultrasonography also provided information on joint features, OA severity, detection of bursitis, synovial effusion, and IA-free bodies [18].

Each of the two treatment sessions consisted of an IA injection of either HA 4 ml (two syringes, 60 mg) or 2% mepivacaine 4 ml. Hyalubrix^® ^is a sterile, nonpyrogenic, viscoelastic solution manufactured with HA sodium salt obtained by bacterial fermentation from a fraction of high molecular weight (>1,500 kDa). Mepivacaine 2%, a local anesthetic, is a sterile isotonic solution manufactured with mepivacaine hydrochloride 20 mg/ml. For the IA injection, the patient was supine with the hip internally rotated 15 to 20°. A 3.5-MHz convex transducer (MyLab25, Esaote, Genova, Italy) fitted with a sterile biopsy guide was used to perform ultrasound guidance. The hip joint was examined by ultrasound using an antero-para-sagittal approach, lateral with respect to femoral vessels defined by color Doppler imaging. The guide was aligned with the long axis of the femoral neck, including the femoral head and the acetabulum. The IA injection was performed by inserting a 20-guage, 0.9 × 90 mm spinal needle into the biopsy guide. Under real-time guidance, the needle was advanced into the anterior capsular recess at the level of the femoral head. After contact with the femoral head, the needle was retracted 1 mm. The medication solution was injected, and the IA localization was monitored by real-time ultrasound visualization and Doppler signal.

### Statistical analysis

All treated patients were considered for statistical analysis. No subsets of patients were evaluated. Statistical analyses were performed on changes or on absolute values, as applicable, at a 5% level of significance. Analyses were performed using SPSS software (IBM corporation, Armonk, NY, USA). Descriptive statistics were calculated on the basis of demographic and clinical data at baseline. Data were described by the mean, median, standard deviation, range, and frequency. Demographic and clinical data of treatment groups were compared at baseline by a chi-square test or a *t *test.

All efficacy variables were analyzed with tests for repeated measures. Differences in VAS pain, NSAID intake, days of intake of NSAIDs in the past month, and global assessment were investigated by an analysis of the variance for repeated measures, whereas differences in Lequesne's algofunctional index were analyzed by a Wilcoxon test. Adverse events that occurred during the study were listed individually.

## Results

Patients' characteristics are presented in Table 1. There were no differences between groups; in the global population, the mean age was 70 ± 8.9 years with an age range of 42 to 79 years, the mean height was 171 ± 7.4 cm, and the mean body mass index was 25.7 ± 3.2. Patients were affected by generalized OA (45%), idiopathic hip OA (95.5%), and localized hip OA (55%). The most frequent Kellgren-Lawrence radiological grade was 3 (85.7%); it was grade 4 in two cases, both randomized to the local anesthetic group.

**Table 1:** Patients' characteristics: demographical, physical and clinical information about patients in study.

Characteristic	Hyalubrix^® ^group (n = 22)	Carbocaine group (n = 20)	Global population (n = 42)
Males	12	10	22
Females	10	10	20
Smokers	4	2	6
Age	68 ± 10.3	67 ± 7.2	70 ± 8.9
Age range	44 to 79	42 to 77	42 to 79
Height	170 ± 6.9	172 ± 8.0	171 ± 7.4
Body mass index	25.6 ± 2.2	24.8 ± 4.1	25.7 ± 3.2
Hip osteoarthritis			
Unilateral right	6 (27.3%)	12 (60.0%)	18 (42.8%)
Unilateral left	4 (18.2%)	1 (5.0%)	5 (10.4%)
Bilateral	12 (54.5%)	7 (35.0%)	19 (45.2%)
Type			
Primary	21 (95.5%)	17 (85.0%)	38 (90.5%)
Secondary	1 (4.5%)	3 (15.0%)	4 (9.5%)
Systemic osteoarthritis			
Yes	11 (50.0%)	8 (40.0%)	19 (45.2%)
No	11 (50.0%)	12 (60.0%)	23 (54.8%)
Duration of osteoarthritis (years)			
Mean	4	5.5	4.71
Median	3.5	3.5	3.5
Standard deviation	2.87	5.1	3.93
Minimum	1	1	1
Maximum	10	20	20
Radiological grade^a^			
2	1 (4.5%)	3 (15.0%)	4 (9.5%)
3	21 (95.5%)	15 (75.0%)	36 (85.7%)
4	-	2 (10.0%)	2 (4.8%)
Knee osteoarthritis			
Absent	12 (54.5%)	12 (60.0%)	24 (57.1%)
Unilateral right	1 (4.5%)	1 (5.0%)	2 (4.8%)
Unilateral left	2 (9.1%)	1 (5.0%)	3 (7.1%)
Bilateral	7 (31.8%)	6 (30.0%)	13 (31%)

Data presented as *n *(%) or mean ± standard deviation unless otherwise stated. ^a^Demographical, physical and clinical conditions of patients in study were similar among two subgroups.

**Figure 1:**
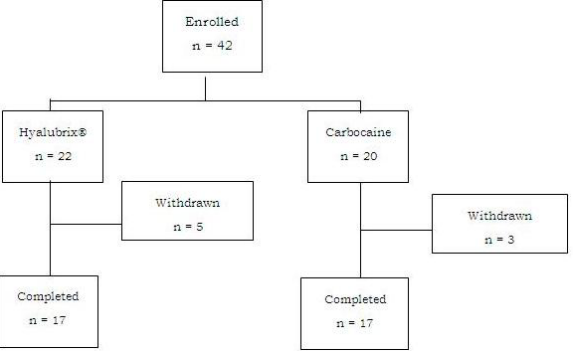
Patients' disposition and study progress.

There were eight (19%) discontinuations, five in the Hyalubrix^® ^treatment group and three in the mepivacaine group (Figure 1), there was one treatment failure per group, and four patients were lost to follow up or retracted their consent to participate. Two patients were not evaluated for the 6-month follow-up visit since comorbidities appearing in the fourth month of the study made correct and exact evaluation of primary and secondary study endpoints impossible; in particular, one patient reported Herpes Zoster virus infection and a need for NSAIDs for symptom control, and one patient reported intense pain for valgus hallux that strongly increased the Lequesne index value and NSAID consumption, both in the Hyalubrix^® ^group.

### Lequesne's algofunctional index

**Figure 2:**
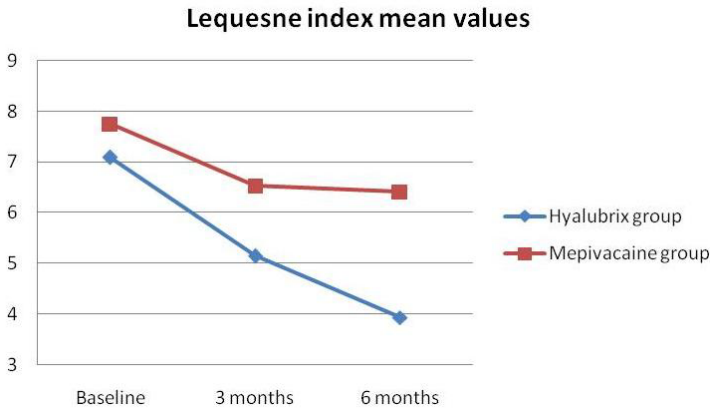
Lequesne index mean values for subgroups of patients treated with Hyalubrix^® ^or mepivacaine via intra-articular ultrasound-guided injection of the hip. P < 0.001 for every value obtained at 3 or 6 months for both subgroups when compared with baseline values. p 0.001 was observed also when comparing values obtained from the hyalubrix^® ^group with those from the mepivacaine group at 3 months, while P < 0.05 was observed when comparing values obtained from the Hyalubrix^® ^group with those from the mepivacaine group at 6 months.

Both treatment groups improved versus baseline at 3 and 6 months (*P *< 0.001). HA was superior to mepivacaine at 3 months (*P *< 0.001) and at 6 months (*P *< 0.05) (Table 2 and Figure 2).

**Table 2:** Lequesne index values for patients undergoing Hyalubrix^® ^or mepivacaine ultrasound-guided intra-articular injection of the hip

Patient subgroup	Lequesne index		
	
	Baseline	3 months	6 months
Hyalubrix^® ^group	7.09 ± 3.78	5.15 ± 5.15^a^	3.94 ± 2.58^a^
Mepivacaine group	7.75 ± 4.15	6.53 ± 4.33	6.41 ± 4.14

Data presented as mean ± standard deviation. All values obtained at 3-month and 6-month control visits reached statistical significance when compared with baseline values. ^a^Values obtained at 3 months were also significantly different when comparing Hyalubrix^® ^results with mepivacaine results in terms of the Lequesne index (*P *< 0.001); at 6 months the significant difference between the two subgroups was still present (*P *< 0.05).

### Secondary outcomes

#### Pain intensity

Both groups improved versus baseline at 3 and 6 months (*P *< 0.001) in both groups (Table 3). HA was superior to mepivacaine for the VAS at 3 and 6 months (*P *< 0.05).

**Table 3:** Secondary outcome measures obtained for subgroups of patients

Secondary outcome measure	Patient subgroup	Baseline	3 months	6 months
Pain visual analogic scale	Hyalubrix^® ^group	6.4 ± 1.94	4.3 ± 2.58^a^	4.5 ± 1.96^a^
	Mepivacaine group	6.0 ± 1.34	4.5 ± 2.63	5.0 ± 2.41
NSAID consumption	Hyalubrix^® ^group	8.5 ± 3.6	2.1 ± 0.4	1.5 ± 0.5
	Mepivacaine group	6.9 ± 2.2	5.5 ± 3.0	2.3 ± 1.0
Global physician assessment	Hyalubrix^® ^group	5.5 ± 1.58	4.4 ± 1.49	4.0 ± 1.51
	Mepivacaine group	5.1 ± 1.41	4.5 ± 1.61	4.3 ± 1.61
Global patient assessment	Hyalubrix^® ^group	6.1 ± 2.07	4.5 ± 2.31	4.0 ± 2.06
	Mepivacaine group	5.7 ± 1.68	4.7 ± 2.33	4.9 ± 2.01

All values reached statistical significance when compared with baseline values ^a^Pain visual analogic scale showed statistical significance also when comparing values obtained in the two subgroups of therapy (*P *< 0.05). NSAID, nonsteroidal anti-inflammatory drug.

#### Nonsteroidal anti-inflammatory drug intake

Both groups improved at 3 and 6 months versus baseline (*P *< 0.001), with no statistically significant differences between treatment groups (Table 3). Even if statistical significance was not reached when comparing results from the Hyalubrix^® ^and local anesthetic groups, the reduction in NSAID intake was 49.4% after 3 months in the Hyalubrix^® ^group and 24.6% in the local anesthetic group.

#### Global assessment

Similar to the change in NSAID intake, there was a significant reduction in patient and physician global assessment from baseline at both 3 and 6 months in both groups (*P *< 0.001), without significance differences between treatment groups (Table 3).

### Tolerability

Two adverse events were reported during the treatment phase. One HA-treated patient experienced moderate hip pain after the second injection that resolved within 7 days of treatment with paracetamol 2 g/day. One mepivacaine-treated patient had mildly intense injection site pain after the first injection, which lasted 36 hours without therapy after the second injection.

No serious adverse events were reported during the study.

## Discussion

The present report describes the first comparative, prospective, double-blind, and randomized study comparing the safety and efficacy of Hyalubrix^® ^with local anesthetic in symptomatic hip OA. The variables registered during the trial were the Lequesne's algofunctional index, the VAS pain score, NSAID intake, and patient's and physician's global assessment as suggested by Outcome Measures in Rheumatoid Arthritis Clinical Trials-Osteoarthritis Research Society International criteria [20]. Of the 42 patients enrolled in the study, the most frequent Kellgren-Lawrence radiological grade was grade 3 (85.7%), and the severest grade 4 was present only in two cases. This result illustrates that visco-induction can be efficacious even in patients presenting a moderate-severe radiological score of hip joint OA.

Although all intra-group differences in efficacy outcomes were statistically significant compared with baseline, there were no significant inter-group differences in NSAID intake or patient's and physician's global assessment at any time during the follow-up. Statistically significant differences between treatments were observed for Lequesne's algofunctional index and the VAS pain score, with patients enrolled in the Hyalubrix^® ^group showing improved scores at 3 and 6 months. Furthermore, with respect to the VAS pain score, the results of this study suggest that pain relief obtained with Hyalubrix^® ^persists for at least 6 months after treatment.

Clinical studies that have examined the efficacy of HA products for hip OA include two systematic reviews [21,22], two randomized controlled trials [23,24], and two uncontrolled trials [25,26]. Among the randomized controlled trials, the study performed by Qvistgaard and colleagues randomized patients into three treatment groups: HA, corticosteroids, and saline solution [23]. The authors concluded that corticosteroids were more effective in relieving pain and improving functionality than HA, which showed good efficacy but did not achieve statistically significant amelioration of symptoms. In contrast, our data suggest that IA HA injection has statistically significant effects on pain and functionality in hip OA-affected patients.

This apparent discrepancy might be explained by some differences between the two studies. First, the follow-up time in our study was longer (6 months versus 3 months), which could facilitate the discovery of long-lasting effects of HA-based therapy. Moreover, Qvistgaard and colleagues administered a total of 60 mg HA (three injections), whereas we administered a total of 120 mg HA (two injections). Different doses of HA might lead to different levels of saturation of CD44 and of other HA receptors such as hyaloadherins, and this might influence receptor activation and biological effects [27]. Also, different molecular weights of HA might lead to different efficacy and safety results. The Hyalubrix^® ^used in this study had a molecular weight >1,500 kDa, whereas the HA product in Qvistgaard and colleagues' study had a molecular weight of 500 to 730 kDa. Furthermore, Qvistgaard and colleagues' compared HA with corticosteroids instead of with local anesthetics.

In the other randomized controlled trial, Tikiz and colleagues compared the efficacies of IA injections of a lower-molecular-weight hyaluronan (HA) and of a higher-molecular-weight visco-supplement in hip OA [24]. No significant differences in outcomes were found between any of the measurements at the first, third, and sixth months.

Other studies [25,26,28-33] with a total of 141 participants treated with one to three injections of Hylan GF20 (Genzyme, Cambridge, MA, USA), reported an overall success rate of approximately 50% after 3 to 12 months. These smaller open-label trials of 10 to 57 participants, without a control group, generally reported moderate improvements in pain and function after treatment with Durolane [28], with Ostenil [26,33], with or Synvisc [25,32]. All studies measured pain (VAS or numerical pain rating scale) or disability (Western Ontario Mac Master scale or Lequesne scale). Because there was no control group and no random allocation, these results must be interpreted with caution, with greater weight placed on the evidence from systematic reviews and randomized controlled trials.

In the present study, global assessments decreased strongly for both local anesthetic and Hyalubrix^®^, and these effects were more evident after 6 months. This result confirms the findings of previous studies of hip OA and of knee OA that HA visco-supplementation is more effective in the long term [8-11].

NSAID consumption in NSAID-taking patients decreased from baseline during the study period. For the group that received Hyalubrix^® ^therapy, however, the NSAID consumption rate was higher at baseline than that of patients treated with local anesthetic. Interestingly, the reduction in NSAID intake was 49.4% after 3 months in the Hyalubrix^® ^group and 24.6% in the local anesthetic group. An NSAID consumption rate decrease and a Lequesne index improvement might have economic impacts. We hypothesize that a reduction in NSAID consumption would lead not only to lesser pharmacological costs for NSAIDs, but also might lead to decreased consumption of other drugs, such as proton pump inhibitors and other medications needed to counteract side effects of NSAIDs. An improvement in the Lequesne index might also lead to improvements in common activities of the patient, such as work (with productive gain) and self-care (with reduced assistance-related costs). These results encourage pharmacoeconomic studies to establish precisely the cost-effectiveness of IA treatment in the management of hip OA.

The low incidence of adverse events (<5%) in the present study of patients undergoing ultrasound-guided IA injection was similar to the safety results obtained by Tikiz and colleagues by fluoroscopic guidance [24], which suggests that ultrasound guidance achieves successful IA injection of the medication. Moreover, the ultrasound-guided technique might be helpful to reduce adverse events related to misplacement of HA during the injection. In addition, the ultrasound-guided technique helps reduce radiation exposure for both patients and clinical operators. The volume of 4 ml drug was well tolerated by all patients when injected into the hip joint.

The present study has a couple of limitations. First, the study did not compare HA treatment with a placebo. The decision to inject local anesthetic instead of placebo was made for ethical reasons; the local ethics committee requested a valid treatment for both groups of patients. Furthermore, a placebo that consists of physiological solution might appear to have a therapeutic effect resulting from the dilution of proinflammatory cytokines. A true placebo would consist of puncture without injection of any solution, but for obvious ethical reasons this experiment could not be performed. Because the present study showed good efficacy of IA injection of local anesthetic in the treatment of hip OA, further studies on the efficacy of this treatment are needed. The number of patients and the follow-up times are sufficient to establish results and significances, but a larger study population and longer follow-up times would increase the certainty of results.

## Conclusions

The present comparative study and previous reports agree on the positive effects of IA HA injection in the management of hip OA. Furthermore, our data suggest that ultrasound-guided visco-induction should be considered a therapeutic option for patients affected by hip OA.

## Abbreviations

HA: hyaluronic acid; IA: intra-articular; NSAID: nonsteroidal anti-inflammatory drug; OA: osteoarthritis; VAS: visual analog scale.

## Competing interests

Fidia Farmaceutici S.p.A. (Padova, Italy) is currently financing the article-processing charge.

## Authors' contributions

All authors equally contributed to the development of study, in gathering and analyzing the data, and in the writing and editing processes of the study.
